# Health-care Resource Requirements and Potential Financial Consequences of an Environmentally Driven Switch in Respiratory Inhaler Use in England

**DOI:** 10.36469/001c.26113

**Published:** 2021-09-23

**Authors:** Darsuh Attar-Zadeh, Harriet Lewis, Martina Orlovic

**Affiliations:** 1 North Central London Clinical Commissioning Group (Barnet Borough), London, United Kingdom; 2 Chiesi Limited, Manchester, United Kingdom; 3 Chiesi Farmaceutici S.p.A, Parma, Italy

**Keywords:** cost, switching, england, national health service, copd, asthma

## Abstract

**Background:** To reduce greenhouse gas emissions, national initiatives advocate the phasing down of respiratory inhalers that use a fluorinated gas as a propellant (pressurised metered-dose inhalers [pMDI]). Nevertheless, pMDIs continue to be an effective and common choice.

**Objective:** To assess the potential financial impact of patients with asthma or chronic obstructive pulmonary disease (COPD) switching from pMDIs to dry powder inhalers (DPIs) in a representative primary care network (PCN) population of 50 000 and the English National Health Service (NHS).

**Methods:** Epidemiological data were combined with current inhaler use patterns to estimate the resources and costs associated with this transition, varying patient acceptance scenarios.

**Results:** Depending on the approach, resource requirements ranged from £18 000 – £53 000 for a PCN, and from £21 – £60 million for the English NHS.

**Discussion:** Significant funds are needed to successfully manage targeted inhaler transitions, together with counselling and follow-up appointment with an appropriately skilled clinician to assess the patient’s inhaler technique and ensure disease control.

**Conclusions:** Targeted transition of inhalers must achieve a balance between environmental impacts, organisational factors, and patient requirements. The resources for managing a switch can be substantial but are necessary to appropriately counsel and support patients, whilst protecting the environment.

## BACKGROUND

Recognising that world-wide emissions of certain substances can significantly deplete and otherwise modify the ozone layer in a manner that is likely to result in adverse effects on human health and the environment, the Montreal Protocol was created in 1987 with the aim of reducing the production and consumption of ozone-depleting substances (“greenhouse” gases).^1^ Adoption of the Montreal Protocol was responsible for an almost 100% replacement of chlorofluorocarbons (CFCs) by hydrofluorocarbons (HFCs), which are not ozone-depleting substances and have a lower global warming potential (GWP).

This initiative led to the development of novel drug delivery systems of inhaled therapies for asthma and chronic obstructive pulmonary disease (COPD), including moving away from CFC-containing metered-dose inhalers (MDIs) to using HFCs as propellants as well as other non-propellant based inhalers such as soft mist inhalers and dry powder inhalers (DPIs). Subsequently, in 2016, the Kigali Amendment was introduced, which centred around the phase-down of HFCs, with the aim of encouraging the use of lower GWP alternatives, where they exist and are available, across several industrial sectors.^2^ With the adoption of the recent Kigali Amendment, the parties involved in enacting the Montreal Protocol have joined the urgent global effort to modulate climate change.

Pressurised MDIs (pMDIs) using HFC propellants nevertheless continue to offer benefits for delivering a wide range of key therapeutic classes of inhaled drugs for the treatment of both asthma and COPD, independent of inspiratory effort by the patient. In the United Kingdom, pMDIs account for a significant proportion (60% to 70%) of inhaler use,^3^ compared with approximately 50% in the rest of Europe.^3–5^ The widespread use has been attributed to the role of short-acting β-agonists as reliever therapy for acute or symptomatic asthma.^6,7^

The carbon footprint contributed by pMDIs represents almost 4% of the total footprint created by the UK National Health Service (NHS).^8^ As a result, health-care practitioners have been incentivized via national targets to reduce prescribing of pMDIs to reduce overall greenhouse gas emissions.^8^

Initiatives targeting the high GWP of fluorinated gases (F-gases) utilised by various industrial sectors have formed a key component of the NHS Sustainable Development Unit strategy to reduce the use of natural resources in health and social care, and to deliver a net zero carbon footprint health service.^8–10^ In Great Britain, the F-Gas Regulation (EU) 517/2014 (as it applies as retained EU law, and as it applies in Northern Ireland directly) specifically mandates a phase-down in the use of HFCs for different sectors. An exemption is granted within the mandate to allow pharmaceutical use of newer hydrofluoroalkane (HFA) propellants until an alternative low GWP propellant has been identified. In the United Kingdom, the introduction of targets for more restrictive use of pMDIs has steadily increased, supported by numerous evaluations of the benefits in reducing the GWP associated with transitioning patients from pMDIs to alternative devices.^11–14^

However, limited consideration has been given to the full costs for balancing such environmental goals against maintaining patient health and well-being, with patient and clinical practice-based elements related to device transition remaining largely overlooked.^15^ Ignoring these aspects ignores the complexity of the decision to switch when taking into account patient preferences and abilities, as well as the economic consequences and operational processes of initiating wholesale transitions to lower carbon options.

Indeed, the intricacy of switching patients between inhaler devices, such that the risks of unintended clinical consequences are minimised, is well-established.^16,17^ Accordingly, close collaboration between health-care practitioners and patients has been identified as a fundamental requirement for transitioning patients, where appropriate, to lower carbon, clinically equivalent inhaler options without compromising their standard of care.^8,10,12,18,19^ The English National Institute of Health and Care Excellence has also highlighted the importance of inhaler choice based on ease of use by the patient.^20^ More than 6 million people in the United Kingdom have some form of chronic lung disorder, most commonly asthma and COPD.^21^ Most of these patients are receiving their therapy via some form of inhaler. Given the relatively high use of pMDIs in the United Kingdom, individual patient discussion and appropriate follow-up is warranted for transitioning patients between inhalers while maintaining their standard of care.^18^

However, an assessment of the resource implications to the NHS to facilitate inhaler device switch from pMDIs to DPIs has yet to be made. Whilst it may be argued that such a switch may result in cost savings due to lower acquisition costs,^14^ implementing policy change is always associated with additional administrative costs to manage such a change, in this case particularly, the additional burden associated with patient counselling and education regarding the device use.^22^

## Objectives

The present study evaluated the potential implications, in terms of service and health-care resource impacts to NHS primary care networks (PCNs) in England, of an environmentally driven policy encouraging the transition of patients with asthma or COPD from pMDI to DPI devices, with a switch to a comparable treatment. The study extrapolated results to a national level to demonstrate potential implications should the transitions occur across the whole English NHS health-care system.

## METHODS

By May 2020, the majority of General Practices in England had formed around 1250 geographical PCNs, covering populations of approximately 30 000–50 000 patients each.^23^ A desk-based analysis of the transition from pMDIs to DPIs for people with asthma and COPD was therefore performed from the perspective of a hypothetical PCN of 50 000 patients in the English NHS. The analysis takes into account direct health-care costs based on personnel costs and unit of resources for managing the switch. NHS staff costs were derived from pay rates from Personal Social Services Research Unit community-based unit costs of health-care professionals (2018/2019).^24^ Drug costs are not considered in the scope of this analysis.

A baseline estimate of the target patient population was made, based upon:

The size of the national population in June 2020;^25^

The epidemiology of asthma and COPD,^25^ including those with dual diagnosis (overlap syndrome);^9,26^ and

The current pattern of inhaler use.^27^

Data on local prevalence and patterns of inhaler use were based on national estimates to calculate the needs of the hypothetical PCN.^28^ The results were also extrapolated to the national level to facilitate estimates representative of England as a whole. To avoid double counting of patients who were diagnosed as having both asthma and COPD, rates of concurrent diagnoses were based on a Clinical Practice Research Datalink study conducted in the United Kingdom that demonstrated that 14.8% of patients with validated asthma had a concurrent COPD diagnosis.^26^

To assess the resource and financial impacts of facilitating a blanket transition of patients from pMDIs to DPIs in clinical scenarios where both inhaler types are appropriate, the analysis considered three hypothetical scenarios. Each considered a different level of patient support provided by practitioners, to ensure operation of the DPI for sufficient inspiratory flow (Table 1):

**Table 1.** Resource Inputs for Each Type of Service Model

**Variable**	**Resource Input**
**Model 1**	
Time spent managing the switch, minutes	5
Pharmacist costs per hour, £	54.00
Model 1: Patients nonadherent, %	0
Model 1a: Patients nonadherent, %	20
Model 1b: Patients nonadherent, %	40
GP costs per consultation, £	39.23
**Model 2**	
Time spent managing the switch, minutes	5
Pharmacist costs per hour, £	54.00
Practice nurse costs per hour, £	42.00
Pharmacist inhaler counselling, %	25
Practice nurse inhaler counselling, %	75
Proportion of patients nonadherent, %	60
Follow-up appointment, pMDI cohort only, minutes	15
**Model 3**	
Time spent managing the switch, minutes	15
Pharmacist costs per hour, £	54.00
Practice nurse costs per hour, £	42.00
Pharmacist inhaler counselling, %	25
Practice nurse inhaler counselling, %	75
Proportion of patients nonadherent, %	10
Follow-up appointment, pMDI cohort only, minutes	15

Abbreviations: GP, general practitioner; NHS, National Health Service; pMDI, pressurised metered-dose inhaler. All costs are sourced from NHS Unit Costs of Health and Social Care 2019.^24^

**Model 1** – A “minimal service” transition conducted by writing to patients to invite them to switch from a pMDI to a DPI, assuming 80% acceptance and 20% requesting a General Practitioner (GP) appointment due to not being comfortable with the required change. An additional scenario was examined with 40% of patients requesting a follow-up GP appointment. Follow-up meeting as a part of the “minimal service” model is not foreseen.

**Model 2** – An “opportunistic” approach, in which patients are counselled to switch from a pMDI to a DPI during an annual review appointment^20^ and some will have a follow-up appointment after 1 month. It was assumed that a clinical pharmacist and practice nurse will require 5 minutes per patient to manage the appointment. A scenario was conducted in which 60% of patients receive a 15-minute follow-up to assess disease control and address any patient concerns about the new device.

**Model 3** – A “gold standard” approach, whereby suggested changes are made depending on a person’s ability to use a particular device. Device switching is managed and counselled by clinical pharmacists and practice nurses, whereby patients attend a targeted initial appointment plus a follow-up appointment after 1 month to be appropriately trained to use the alternative inhaler device. Such an approach could be considered to be the “gold standard” for providing patient support on inhaler training.^29^

## RESULTS

The use of pMDIs only was estimated to be 71% of patients with asthma and 39% of those with COPD, while 3% of patients with asthma and 16% with COPD, respectively, used both pMDIs and DPIs.^27^ Using the prevalence data based on Quality and Outcomes Framework data for 2018-19^9^ and the population of England from the Office for National Statistics,^25^ the estimated population with asthma or COPD, adjusting for a concomitant asthma and COPD diagnoses, is approximately 3.9 million people (Table 2). The corresponding population size of a hypothetical PCN (50 000 population) is approximately 3542 patients. The estimated population size that would be targeted for the device switch was 2.1 million asthma and 0.6 million COPD patients in England and 1905 asthma and 532 COPD patients in a hypothetical PCN (Table 2).

**Table 2.** Epidemiological and Inhaler Usage Estimates

	**Asthma**	**COPD**
**Epidemiological Parameters**		
Prevalence, %^9^	6.05	1.93
**Population of England, n^25^**	56 287 000	56 287 000
Concomitant asthma and COPD diagnosis, %^26^	14.8	
Adjusted prevalence for ACOS, %	5.15	1.93
**Population Estimates**		
Patients in England, n	3 405 364	1 086 339
Patients in a 50 000 population PCN, n	3025	965
ACOS adjusted patient numbers for England, n	2 901 370	1 086 339
ACOS adjusted patient numbers per 50 000 patients, n	2577	965
**Inhaler Usage Parameters** ^27^		
pMDIs only, %	71	39
pMDIs only in England, n	2 069 796	425 498
pMDIs only per 50 000 patients, n	1839	378
pMDIs and DPIs, %	3	16
pMDIs and DPIs in England, n	75 002	173 658
pMDIs and DPIs per 50 000 patients, n	67	154

Abbreviations: ACOS, asthma-COPD overlap syndrome; DPI, dry powder inhaler; PCN, primary care network; pMDI, pressurised metered-dose inhaler.

Each evaluated model has different resource implications for both the hypothetical PCN cohort and extrapolation to the population of England (Table 3). In Model 1, if all patients accept the switch mandated via letter, the cost of the model would be £10 969 for the PCN or £12 347 793 at an NHS England level. Further, the overall costs for Model 1 with minimal service input, but including 20% of patients who would not be satisfied with a mandated change and would require further counselling was £30 093 at a PCN level or £33 876 855 at an NHS England level (Table 3, Model 1). Increasing the proportion of patients who would require follow-up appointments can substantially increase the costs. For example, increasing the proportion of dissatisfied patients to 40% increased management costs by 64%.

**Table 3.** Cost Estimates: Model 1 (“minimal service input”)

**Area**	**Asthma Counselling (£)**	**COPD Counselling (£)**	**Nonadherent Patients, n**	**Additional GP Appointments Costs (£)**	**Total (£)**	**Total (£)**
**Model 1: 0% of Patients Nonadherent, Requiring Additional Counselling**						
pMDIs only – England	9 314 082	1 914 739	-	-		12 347 793
pMDIs and DPIs – England	337 508	781 463	-	-		
pMDIs only – 50,000 patients PCN	8274	1701	-	-		10 969
PMDIs and DPIs – 50,000 patients PCN	300	694	-	-		
**Model 1a: 20% of Patients Nonadherent, Requiring Additional Counselling**						
pMDIs only – England	9 314 082	1 914 739	499 059	19 578 073	30 806 894	33 876 855
pMDIs and DPIs – England	337 508	781 463	49 732	1 950 989	3 069 961	
pMDIs only – 50,000 patients PCN	8274	1701	443	17 391	27 366	30 093
PMDIs and DPIs – 50,000 patients PCN	300	694	44	1733	2727	
**Model 1b: 40% of Patients Nonadherent, Requiring Additional Counselling**						
pMDIs only – England	9 314 082	1 914 739	998 117	39 156 146	50 384 967	55 405 917
pMDIs and DPIs – England	337 508	781 463	99 464	3 901 978	5 020 950	
pMDIs only – 50,000 patients PCN	8274	1701	887	34 783	44 757	49 217
PMDIs and DPIs – 50,000 patients PCN	300	694	88	3466	4460	

Abbreviations: DPI, dry powder inhaler; PCN, primary care network; pMDI, pressurised metered-dose inhaler.

**Table 3.** Cost Estimates: Model 2 (“opportunistic switch”)

**Area**	**Asthma Counselling (£)**	**COPD Counselling (£)**	**Additional Counselling Asthma (£)**	**Additional Counselling COPD (£)**	**Total (£)**	**Total (£)**
**Model 2a: 10 Minutes Spent Counselling**						
pMDIs only – England	15 523 470	3 191 232	-	-	18 714 702	20 597 654
pMDIs and DPIs – England	562 514	1 302 439	-	-	1 864 953	
pMDIs only – 50,000 patients PCN	13 790	2835	-	-	16 624	18 281
PMDIs and DPIs – 50,000 patients PCN	500	1157	-	-	1657	
**Model 2b: 60% of Patients Require Additional 15 Minutes Follow-up Counselling**						
pMDIs only – England	15 523 470	3 191 232	13 971 123	2 872 109	35 557 933	59 680 997
pMDIs and DPIs – England	562 514	1 302 439	506 263	1 172 195	3 543 410	
pMDIs only – 50,000 patients PCN	13 790	2835	12 411	2551	31 586	53 015
PMDIs and DPIs – 50,000 patients PCN	500	1157	450	1041	3148	

Abbreviations: DPI, dry powder inhaler; PCN, primary care network; pMDI, pressurised metered-dose inhaler.

**Table 3.** Cost Estimates: Model 3 (“Gold standard”)

**Area**	**Asthma Counselling (£)**	**COPD Counselling (£)**	**Additional Counselling Asthma (£)**	**Additional Counselling COPD (£)**	**Total (£)**	**Total (£)**
**Model 3: All Patients Receive Dedicated Appointment with Pharmacist and Follow-up Counselling**						
pMDIs only – England	23 285 204	4 786 848	23 285 204	4 786 848	28 072 052	30 869 481
pMDIs and DPIs – England	843 771	1 953 658	843 771	1 953 658	2 797 429	
pMDIs only – 50,000 patients PCN	20 684	4252	20 684	4252	24 937	27 422
PMDIs and DPIs – 50,000 patients PCN	750	1735	750	1735	2485	

Abbreviations: DPI, dry powder inhaler; PCN, primary care network; pMDI, pressurised metered-dose inhaler.

Following “opportunistic” counselling in Model 2, the overall costs were £18 281 at a PCN level, or £20 579 654 at an NHS England level for patients whose switch is conducted during an annual review appointment. For patients who required additional follow-up counselling, the costs increased to £53 015 at a PCN level and £59 680 997 at a national level (Table 3, Model 2). Since many patients require a check of the inhaler technique and assessment of disease control, it is expected that a significant proportion of patients who would require additional follow-up meetings would require minimal time (5 minutes) spent describing the new device technique, assessing the patient’s ability to use the new device, and explaining why the change was initially recommended.

**Figure 1.**
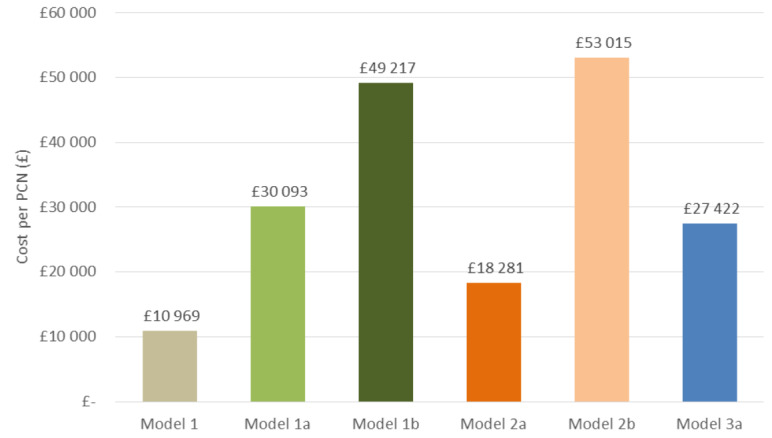
Model Costs for Hypothetical PCN Model 1: 0% of patients nonadherent, requiring additional counselling Model 1a: 20% of patients nonadherent, requiring additional counselling Model 1b: 40% of patients nonadherent, requiring additional counselling Model 2a: 10 minutes spent counselling Model 2b: 60% of patients require additional 15 minutes follow up counselling Model 3: All patients receive dedicated appointment with pharmacist and follow up counselling

Finally, using the “gold standard” approach, in which patients received initial and dedicated follow-up appointments and counselling with a pharmacist or practice nurse, the overall cost to the PCN was estimated to be £27 422, and £30 869 481 when extrapolated to the NHS England level (Table 3, Model 3). This approach, the most patient-centric, could offer the optimal efficacy outcome at a reasonable overall cost. Costs of all scenarios for a hypothetical PCN are shown in Figure 1.

## DISCUSSION

Using a range of modelled scenarios, this analysis illustrates the resource implications and financial consequences of managing the transition of patients from pMDIs to DPIs in a hypothetical PCN, as well as the need for organisational changes and appropriate resource allocation. To the best of our knowledge, this is the first attempt to characterise administrative resource requirements focusing on personnel costs of such a transition for non-clinical reasons. The perspective of a PCN was utilised, as these stakeholders are responsible for the funding and delivery of NHS local services in England, and would be responsible for implementing this policy. Our evaluation suggests that, at all levels of service, any large-scale transition of patients with asthma or COPD from pMDIs to DPIs would have a significant impact on local NHS resources.

With constant pressure to reduce costs and improve effectiveness of limited budgets, it may be challenging to secure additional funds to appropriately support patients through this transition. In Model 1, the follow-up is not proactively done, so if there were any issues or additional questions the patient would contact the practice or community pharmacy. Therefore, assuming Model 1, which represented the lowest-resourced service and involved minimal practitioner support with little or no consideration of the patient preference, the cost to a hypothetical PCN representing 50 000 patients ranged from £10 969 to £49 217, depending on the number of patients not accepting the switch mandated via letter (0%, 20%, or 40% requesting a follow-up). This shows that costs could easily increase with patients seeking additional support and counselling. Patient-centred care is a key NHS commitment to foster the partnership between the patient and health-care system, so it is not likely that this model would be implemented.^30,31^

In the scenario that entailed discussing the switch during the annual asthma review, in accordance with existing National Institute of Health and Care Excellence recommendations^7^ (Model 2), whereby a portion of patients would receive a follow-up appointment, the resulting costs to the hypothetical PCN ranged between £18 281 and £53 015. Lastly, overall costs to a hypothetical PCN were estimated to be £27 422 with the highest level of practitioner–patient interaction that maintains the standard of care of asthma or COPD treatment for the patient, assesses the patient’s ability to use the device and thereby optimises health outcomes. Extrapolating to the full asthma and COPD population in England, these costs would range from £12 - £55 million for Model 1, £21 - £60 million for Model 2 and £31 million for Model 3, depending on the scenario.

The UK government has recommended that low GWP inhalers be promoted within the NHS unless there are specific medical reasons for not doing so, and that the NHS should set a target that at least 50% of prescribed inhalers should be low GWP by 2022.^8^ In January 2019, an expert working group was convened to evaluate potential strategies to achieve this goal.^28,32^ Accordingly, health-care practitioners have now been directed to implement this environmentally driven change. For asthma management, structured medication reviews or planned asthma reviews for all inhaler prescriptions taking place in primary care should consider moving patients to lower-carbon options where clinically appropriate.^17,28^ While this level of counselling is represented in Model 2 of the analysis, it does not consider the full support needed for education and understanding patient preferences that may not be feasible during the 5-minute review appointment.

Hence, in our analysis, Model 3 can be considered the “gold standard” service model, providing an opportunity for patients to be involved in the decision about which inhaler would be most suitable given their abilities and preferences, which would then affect treatment adherence and disease control. It is expected that this model might entail improved and sustained transitions as part of shared decision-making.

There is substantial evidence that suboptimal use of inhalers by patients is already a common problem in asthma and COPD, and patients have preferences for particular inhaler devices that are associated with increased ease of use.^33–35^ In addition, the effect of inhaled therapy is largely dependent on the patient’s preferences and ability to use the inhaler devices correctly.^36^ Only a limited number of patients with asthma or COPD (estimated to be ~30% to 40%) have been observed to use their inhalers correctly, and the frequency of errors has not reduced over the 40-year span of the studies.^37,38^ Up to 45% of patients make at least one error, including 50% related to devices and 31% related to inhalation technique, while 19% make errors related both to devices and to inhalation technique.^39^ Indeed, one study found that in patients using multi-dose DPIs, 46% demonstrated at least one critical error; this rate was 15% for soft mist inhalers, 13% for single-dose DPIs and 8% for pMDIs.^36^

Since inhalers are key to the management of daily symptoms, chronic disease and acute emergencies, the choice of inhaler device and inhaler technique teaching has a major impact on patient outcomes.^40^ Making at least one critical error due to device switch can lead to increased health-care expenditure related to the need for additional health-care visits and pharmacological treatment.^41^ Even without consideration of switching, ongoing verification and proper inhalation technique training in all patients who are regularly treated with inhalers has been deemed necessary for required improved inhaler technique and optimal clinical outcomes in patients with asthma or COPD.^42,43^

All types of inhalers as delivery systems have unique roles in the treatment of asthma and COPD, and no single delivery system can be considered universally acceptable for all patient groups.^40^ It is, therefore, important that the choice of device is tailored to meet individual patients’ needs, preferences, and satisfaction, while offering the requisite level of disease control.^15^ Indeed, choosing an appropriate device according to patient characteristics is one of the primary steps in optimal control of the disease,^44–46^ and is considered to be equally important as using disease severity as a guide to choosing the right medication.^44^

The debate around switching inhalers to reduce the environmental impact of propellants requires a broad view of the impact of such a policy change, specifically with respect to the importance of patients having a choice, as well as the administration and resources required to support the switch to different inhalers.^47^ If policy-makers are justifiably concerned about sustainability and reducing the GWP of health care, it is critical to consider issues other than propellants and plastic waste.

There is potential waste and environmental damage caused by poorly managed asthma and COPD, which leads to reduced work and school productivity, unnecessary use of unscheduled health care with unnecessary trips to hospitals and clinics, and further burden on health-care resources, as well as costly waste of inhalers due to incorrect use. Further, some studies show that overuse of inhalers, especially in asthma, is also contributing to poor disease control^48^ and creating a large unnecessary carbon footprint. These represent resources that would be saved if patients were taught more effective self-management, both for routine treatment and disease exacerbations.^18,49^ This also provides an option that can be further explored to optimize patient management whilst generating positive environmental impact.

HFAs in pMDIs account for approximately 4% of the carbon footprint of the NHS, which in turn accounts for 3.5% of the total UK carbon footprint, meaning that the contribution of pMDIs to the total UK carbon footprint is 0.1%. The development of alternative low GWP propellants, and enhanced recycling schemes, represent important objectives for some manufacturers; however, in the meantime, patients with asthma and COPD should retain access to pMDIs. This may require a temporary exemption for the continued production and use of HFAs for medical purposes, just as there was for CFCs in the 1990s, until newer models with reduced environmental impact, at the same level of DPIs become commercially available.^18,50^

## Limitations

This study had several limitations. The study only considered direct personnel costs related to inhaler switch management and patient counselling. A comprehensive analysis of the relationship between inhaler technique and exacerbation rates was beyond the scope of this study, and the potential impact on patient health-related quality of life (disutility) was also not considered. In addition, health-care resource assumptions were based on a single hypothetical PCN, and it is likely that resource use may differ between PCNs; the variability in cost of this resource use was not included. Furthermore, our estimates of costs were based on the proportions of patients compliant with switching and, whilst we have presented scenarios to account for this, patient behaviour in real life can differ. We also presented data on the assumption that the whole of the eligible population in hypothetical PCN and England would transition—although in real life it is possible that certain subgroups would be prioritised for the switch, and that the costs of switch management could be phased out over several years.

## CONCLUSION

Switching inhaler devices for environmental reasons alone is a complex activity that should be evaluated looking at individual and organisational factors. To date, the approach by policy-makers has been overly simplistic without sufficient consideration of the implications for implementation in a real-world setting, the impact on patient care, and the capacity, capability, and resources for local front-line health-care providers. Given the continuous pressure for efficient use of limited health-care resources, any policy mandating such transition must achieve a balance between environmental impact, patient requirements, organisational factors, and resulting health impacts.

The costs and time implications for managing a switch can be substantial, and it may be difficult to ensure sufficient funds and capacity are available to appropriately counsel and support patients; otherwise, inappropriate medicine optimisation may pose a threat to both patients’ health and the environment. In recognition of the importance of reducing the carbon footprint, the most environment-friendly inhaler is the one that achieves the best clinical outcome for the patient, whilst minimising the need for additional health-care resources.
